# Modeling of the Temporal Patterns of Fluoxetine Prescriptions and Suicide Rates in the United States

**DOI:** 10.1371/journal.pmed.0030190

**Published:** 2006-06-13

**Authors:** Michael S Milane, Marc A Suchard, Ma-Li Wong, Julio Licinio

**Affiliations:** **1**Center for Pharmacogenomics and Clinical Pharmacology, Semel Institute for Neuroscience and Human Behavior, University of California Los Angeles, Los Angeles, California, United States of America; **2**Department of Psychiatry and Biobehavioral Sciences, University of California Los Angeles, Los Angeles, California, United States of America; **3**Department of Biomathematics, David Geffen School of Medicine, University of California Los Angeles, Los Angeles, California, United States of America; **4**Division of Endocrinology, Diabetes and Hypertension, Department of Medicine, David Geffen School of Medicine, University of California Los Angeles, Los Angeles, California, United States of America; James Cook UniversityAustralia

## Abstract

**Background:**

To study the potential association of antidepressant use and suicide at a population level, we analyzed the associations between suicide rates and dispensing of the prototypic SSRI antidepressant fluoxetine in the United States during the period 1960–2002.

**Methods and Findings:**

Sources of data included Centers of Disease Control and US Census Bureau age-adjusted suicide rates since 1960 and numbers of fluoxetine sales in the US, since its introduction in 1988. We conducted statistical analysis of age-adjusted population data and prescription numbers. Suicide rates fluctuated between 12.2 and 13.7 per 100,000 for the entire population from the early 1960s until 1988. Since then, suicide rates have gradually declined, with the lowest value of 10.4 per 100,000 in 2000. This steady decline is significantly associated with increased numbers of fluoxetine prescriptions dispensed from 2,469,000 in 1988 to 33,320,000 in 2002 (*r_s_* = −0.92; *p* < 0.001). Mathematical modeling of what suicide rates would have been during the 1988–2002 period based on pre-1988 data indicates that since the introduction of fluoxetine in 1988 through 2002 there has been a cumulative decrease in expected suicide mortality of 33,600 individuals (posterior median, 95% Bayesian credible interval 22,400–45,000).

**Conclusions:**

The introduction of SSRIs in 1988 has been temporally associated with a substantial reduction in the number of suicides. This effect may have been more apparent in the female population, whom we postulate might have particularly benefited from SSRI treatment. While these types of data cannot lead to conclusions on causality, we suggest here that in the context of untreated depression being the major cause of suicide, antidepressant treatment could have had a contributory role in the reduction of suicide rates in the period 1988–2002.

## Abstract 2

An association has been shown between the introduction of the antidepressant fluoxetine in the USA in 1988 and a reduction in the number of suicides.

## Editors' Summary

**Background.:**

Depression is very common. For example, in the US, an estimated 10% of men and 20% of women will suffer from major depression at some stage in their lives. One way of treating the condition is with drugs. Several types of antidepressant drugs are available, and in many countries they are among the most commonly prescribed medicines. However, all antidepressants have side effects.

One family of antidepressants, called selective serotonin uptake inhibitors (SSRIs), was introduced in the late 1980s. The name of these drugs comes from their effect, which is to prevent the removal (reuptake) from the nerve endings of one type of chemical (serotonin) that is important for transmitting nerve impulses between brain cells. SSRIs are claimed to be more effective and to have fewer side effects than older antidepressants, and many brands of SSRI are now on the market. However, in recent years there have been claims that some people taking SSRIs have committed suicide as a result of the drugs. Whether the SSRIs are the cause of the suicide is hard to know, because people who are depressed do sometimes feel like killing themselves; so if a depressed person taking an SSRI commits suicide, it is hard to tell whether this is a result of the depression or a side effect of the treatment (the SSRI). The drug regulatory authorities in some countries are now carefully studying the issue of suicides and antidepressant use, both in adults and in children. The US Federal Drug Administration has issued what it calls a “black box warning” on the use of these drugs.

**Why Was This Study Done?:**

The researchers wanted to discover whether the number of suicides in the US had increased or decreased since treatment with the first widely used SSRI (fluoxetine, also known as Prozac) began in 1988. Any difference in the number of suicides found before and after that date would not necessarily be the result of the introduction of this antidepressant, or other SSRIs, but the information would provide helpful information about the effects of these drugs.

**What Did the Researchers Do and Find?:**

They looked at annual suicide rates between 1960 and 1988 and compared them with annual rates in the period 1988 to 2002. They used several sources of data, including the Centers of Disease Control and the US Census Bureau. The researchers found that from the early 1960s until 1988, in the entire US population, between 12.2 and 13.7 people in every 100,000 committed suicide each year. After that time, the numbers of suicides gradually declined, with the lowest figure (10.4 people per 100,000) reached in 2000. The researchers did mathematical tests, which demonstrated that the steady decline was statistically associated with the increased number of fluoxetine prescriptions—that is, the more prescriptions there were, the fewer suicides there were. (There were around two-and-a-half million prescriptions of the drug in 1988, increasing to over 33 million in 2002.)

**What Do These Findings Mean?:**

In all scientific research, it is an important principle that finding an association between two events does not prove that one caused the other to occur. However, the authors of this paper suggest that the use of this drug could have contributed to the reduction of suicide rates in the US in the period 1988 to 2002. Several other SSRIs are also now in common use, but they were not considered in this study, nor were other antidepressants, or other treatments for depression.

**Additional Information.:**

As depression is such a common condition—and because there are so many ways of treating it, including counseling and psychotherapy—there are many Web sites devoted to the subject. We have given a small selection below. Please access these Web sites via the online version of this summary at http://dx.doi.org/10.1371/journal.pmed.0030190.

• From the American Academy of Family Physicians (AAFP), general advice on depression

• Also from the AAFP, advice specifically about antidepressant drugs

• MedlinePlus brings together authoritative information about depression from the US National Library of Medicine, National Institutes of Health, and other government agencies and health-related organizations

• Health pages of the BBC on depression

• Information about depression from other UK health advice sites: Patient UK and NetDoctor.co.uk

## Introduction

Because of evidence made available in recent months, US and UK regulatory agencies have been critically examining suicidality and antidepressant use in children and adults. The crucial point is whether antidepressants increase suicidality over and above what is caused by the underlying disorders, such as major depression. With such recent scrutiny of antidepressants, particularly selective serotonin reuptake inhibitors (SSRIs) and the US Food and Drug Administration-recommended “black box warning,” it becomes timely to examine temporal trends in suicide and to study the potential impact of antidepressants on mortality caused by self-harm.

This is a complex task because while on the one hand acute antidepressant use has been associated with suicidality, but untreated depression is also the major cause of suicide [1]. Therefore, two competing hypothesis exist. The first is that the acute effects of antidepressants can induce suicidality, and the second is that by effectively treating depression, antidepressants can reduce the rates of suicide.

Major depressive disorder is a common and complex disorder of gene-environment interactions, for which there is no curative treatment [2–4]. The disorder afflicts approximately 10% of American men and 20% of American women over their lifetimes. The point prevalence is in the range of 3% (2% in men, 4% in women) [5,6], but increases up to 10% in the elderly [7,8]. Because the prevalence of depression is so high, and treatment lasts several months to years, antidepressant pharmacotherapy is among the most frequently used treatments in all of medical therapeutics.

Depression is itself the most prevalent cause of suicides [1], and suicide is, in turn, still among the major causes of death. According to the latest figures from the Centers for Disease Control and Prevention (CDC), in the United States in 2002, suicide was the eleventh leading cause of death (in 1998 it was the eighth leading cause of death). When the data are analyzed by age cohort, suicide is the fifth leading cause of death in the age group 5–14, the third leading cause of death in the age group 15–24, and the fourth cause of death in the age group 25–44 (Table 1) [9]. It has been estimated that 60%–70% of acutely depressed patients experience suicide ideations [10]. It is universally agreed that depression increases the risk for suicide. However, the extent of the risk has been a subject of debate. The figure of lifetime risk for suicide in patients with major depression had been commonly quoted as in the range of 10%–20% [10–12], based on the study of hospitalized patients. However, recent studies have examined other types of samples and found a much lower risk, reported as 6% by Inskip et al. [13] based on a meta-analysis, 3.4%–3.5% (7% in males and 1% in women) based on gender and age-stratified calculations made on the entire population [14,15], and 2.4% based on analysis of a community sample in England [16]. An interesting meta-analysis in which papers were stratified by type of presentation revealed that in patients hospitalized for suicidality the lifetime prevalence of suicide was 8.6%; for affective disorder patients hospitalized without specification of suicidality, the lifetime risk of suicide was 4.0%, and for mixed inpatient/outpatient populations it was 2.2%; for the nonaffectively ill population, it was less than 0.5% [17]. Depression appears to be present in at least 50% of all suicides in adults [18,19]; in children that rate has been reported to be in the range of 62%–76% [20,21].

**Table 1:**
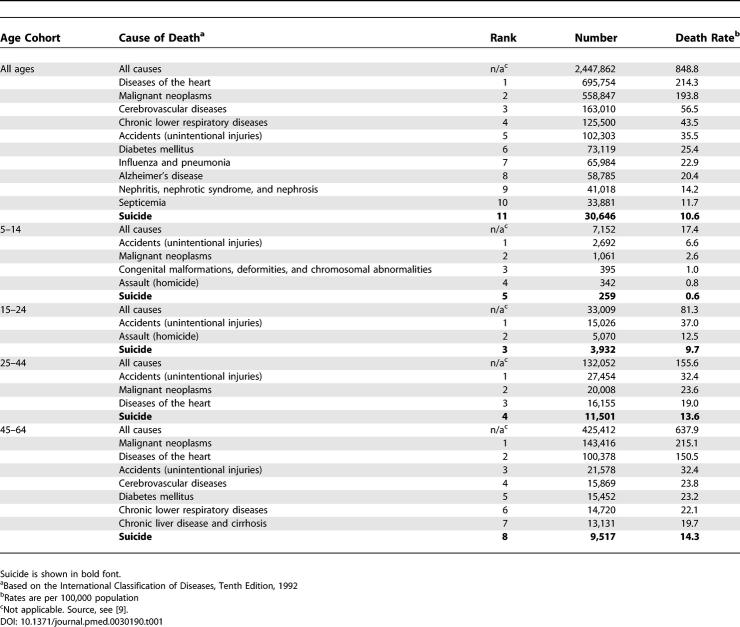
Suicide as Cause of Death in Various Age Cohorts in the United States, 2002

The prototypic SSRI fluoxetine was introduced in the United States in 1988. Since then, fluoxetine became the most widely prescribed antidepressant drug in the world [22]. Since its introduction, fluoxetine has also been approved for use in the treatment of patients with other disorders. It is currently the only antidepressant that is FDA-approved for the treatment of depression in children. Fluoxetine is a selective inhibitor of serotonin reuptake and initially after administration it has limited effect on other neurotransmitters [23]. It is well absorbed after oral administration, with peak plasma concentrations observed after 6–8 h. The parent compound, fluoxetine, has an elimination half-life of 1–4 d as compared to the active metabolite, norfluoxetine, which has a half-life of 7–10 d [24]. This extended half-life appeared to protect against sporadic noncompliance and against the occurrence of withdrawal phenomena [23].

Fluoxetine has been widely studied and described in the scientific literature throughout the years and its use has been reported in over 8,500 articles present in literature databases (Medline and Embase databases until May 2003) [25]. All data from the various meta-analyses confirm that in the treatment of patients with major depression, fluoxetine has been as effective as tricyclic antidepressants, and has shown to have a distinctly more benign side effect profile, be safer in overdose, easier and simpler for patients to use and physicians to prescribe, and to have lower rates of discontinuation [26]. Because of the fact that it was the first SSRI introduced to market and cumulatively the most prescribed, we have used fluoxetine as a prototypical SSRI and studied trends in fluoxetine prescription as a model for SSRI use since 1988.

There are now two competing hypotheses on this important clinical issue: (i) Antidepressants can trigger suicide, or (ii) by treating depression, which is the largest cause of suicide, antidepressants reduce overall suicide rates. We used annual dispensing information on fluoxetine and suicide rates in the United States to address the question of whether antidepressants by themselves are associated with increased suicide numbers, or if they are linked to decreased suicide rates.

## Methods

### Population Data

The total population data and distributions for the years 1960–2002 were obtained from the US Census Bureau. Sources for the 1960–1999 period included Historical National Population Estimates (July 1, 1900 to 2000) from the US Census Bureau [27]. Data for Table 2 come from Current Population Reports [28] and US Census Bureau National Population Estimates web pages (http://www.census.gov/popest/estimates.php). National population data for the years 1960–1979 cover the resident population plus Armed Forces overseas. National population data for all other years cover only the resident population. In addition, sources for the 2000–2002 period include the US Census Bureau's American FactFinder's Data Sets: Population Estimates [29].

**Table 2:**
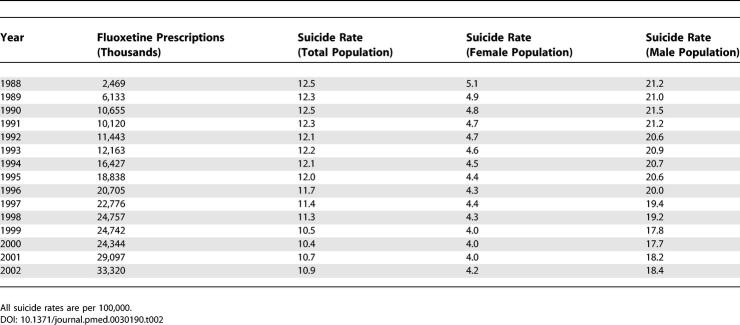
Suicide Rates for the Total, Female, and Male Populations and Fluoxetine Prescription Data for the 1988–2002 Period

### Suicide Data

The mortality data for this study were collected from annual statistical files of the National Vital Statistics System compiled by the CDC, which is a compilation of statistics from all death certificates filed in the 50 states and the District of Columbia [30]. These data are based on the implementation of a new population standard for age standardization (age-adjustment) of death rates by the CDC. The new standard is based on the year 2000 population and has replaced the existing standard based on the 1940 population. This direct and indirect standardization and statistical variability in age-adjusted death rates has provided age-adjusted death rates that are substantially higher than those based on the 1940 standards. Furthermore, the new standards represent more accurate trends in age-adjusted death rates for certain specific causes of death, such as suicide, and have narrowed race differentials in age-adjusted death rates [31].

In previous studies, crude death rates have been a widely used measure of mortality. However, crude death rates are influenced by the age composition of the population. As such, comparisons of crude death rates over time or between groups may be misleading if the populations that are being compared differ in age composition. Age standardization is one of the key tools used to control for the changing age distribution of the population, and thereby to make meaningful comparisons of vital rates over time and between groups. This age-adjusted comparison is free from the confounding effect of changing age distribution and therefore, better reflects the trend in US mortality [31]. The use of age adjustment requires a “standard population,” which is a set of arbitrary population weights. For this purpose, the population age distribution for the year 2000 standards were prepared by the US Bureau of the Census [32] and converted by the National Center for Health Statistics to a standard million population by dividing the age-specific populations by the total population and multiplying by 1 million.

### Fluoxetine Prescription Data

We obtained the estimates for the prescription trends and dispensed numbers for fluoxetine from IMS Health USA, the leading international provider of data on drug use to the pharmaceutical and healthcare industries. The data for fluoxetine were extracted from their extensive National Prescription Audit database in Philadelphia, which provides information based on dispensed prescriptions from retail, mail service, and LTC pharmacies. National Prescription Audit data are projected to a national level from a sample of over 20,000 retail pharmacies. These data are representative of the total dispensed prescriptions for fluoxetine from when it was introduced to the market in 1988–2002. The dispensed numbers were reported in thousands for the formulations Prozac (SMRY 0188 LLY), Prozac Weekly (SMRY 0301 LLY), and fluoxetine-HCl (SMRY 0000 USA). The fluoxetine-HCl generic formulation and Prozac Weekly formulation were first introduced in 2001. The numbers for the Prozac Weekly regimen were multiplied by seven in order to account for the single weekly dispensation of this type of formulation and to adjust for the dispensation numbers in correlation with rest of the daily-dispensed data.

The fluoxetine data on the percent of dispensation for specific disorders were obtained independently of the yearly dispensed data from IMS Health for the period of 2002–2004, with 2002 being the first year that they kept track of this type of information in their database. These data provided a specific list of disorders that fluoxetine was prescribed for and the relative percentage of dispensation of fluoxetine in the given year for these disorders. See Table 2 for suicide rates and fluoxetine prescription data for the period 1988–2002.

### Statistical Analyses and Modeling Methods

We first examined the association between fluoxetine prescription numbers and suicide rates in 1988–2002 using Spearman's rank correlation test. This nonparametric test is more robust than Pearson's correlation coefficient, as the test does not assume that the data come from a bivariate normal distribution.

To predict suicide mortality in 1988–2002, assuming that pre-1988 trends had been maintained and accurately estimated the cumulative decrease in deaths attributable to fluoxetine, we developed a time-series regression model. The model assumes that the underlying time dynamics in age-adjusted suicide rates are described by two effects. The first is an autoregressive (AR) random walk process between 1960 and 2002 and the second is a linear regression model that incorporates fluoxetine prescription numbers as a covariate from 1988 onward. AR processes are standardly employed to capture the dependence between observations in randomly varying time series and to utilize this dependence to make forward predictions [33]. We selected the appropriate order of the AR process and of the fluoxetine effect via model selection, leading to a first-order AR process and linear fluoxetine effect. Under this model, let *Y_t_* equal the age-adjusted suicide rate (measure in percent of deaths) and *X_t_* equal the number of fluoxetine prescriptions (measured in millions) at time *t.*



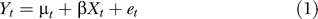



and



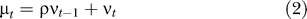



where β is the fluoxetine effect size, ρ is the AR lag-1 parameter, and *e_t_* and *v_t_* are independent Gaussian white noise.

We fit this AR regression model in a Bayesian framework [34], assuming diffuse priors on model parameters. Unlike likelihood-based estimation procedures, Bayesian approaches do not require a stationarity constraint (−1 ≤ ρ ≤ 1) on the underlying random walk process [35]. The assumption of stationarity in this form of regression model would imply that any long-term drift of suicide rates resulted from the additive covariate *X_t_* and were not generated by the AR process. Such an assumption would lead to an unfair test of the influence of fluoxetine on suicide rates if these rates were naturally decreasing over time. Rather, we opted for a potentially nonstationary model. As a consequence, identifiability between the covariate effect and AR coefficients may be compromised [36]. We guarded against this shortcoming by employing suicide rate observations that extend as far back in the past as possible before the introduction of SSRIs. These early time points yield information in our model solely about the underlying random walk characterized by ρ, separating it from a potential fluoxetine effect later in time estimated by β.

To examine potential differences in response to fluoxetine across female and male populations, we refitted our time-series regression model to suicide rates stratified by gender. In this model, we assumed that the AR process was the same for both females and males such that they shared a common AR lag-1 parameter ρ; however, females and males may have different fluoxetine effect sizes, and Δ represents this difference. This approach assumes that female and male prescription rates were the same.

## Results

### Percent of Fluoxetine Dispensed for Depression

By examining the data on fluoxetine dispensation for the specific associated disorders, we found, as expected, that for the period 2002–2004, depression was the primary reason that these drugs were dispensed. Using this sample time period, as an indicator of relative use of these drugs, we found that for the period 2002–2004, major depression was the reason for 72.1% of all fluoxetine that was dispensed, with the other 27.9% dispensed for the following disorders in order of frequency: anxiety (6.2%), obsessive-compulsive disorder (5.7%), schizophrenia (2.2%), stress (2.0%), premenstrual syndrome (1.0%), phobias (0.8%), eating disorders (1.0%), and all others unclassified (8.8%).

### Trends in Suicide and Fluoxetine Dispensing

**Figure 1:**
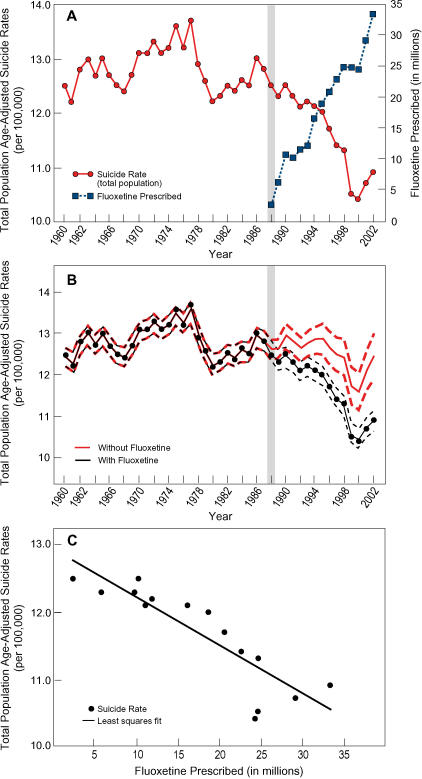
Age-Adjusted Suicide Rates at the Population Level (A) Age-adjusted suicide rates (per 100,000) for the total population from 1960 to 2002 and fluoxetine prescribed numbers (in millions) from 1988 to 2002 (B) Age-adjusted suicide rate predictions for the total population. The solid lines trace out the posterior median model predictions and the dashed lines depict the 95% Bayesian credible intervals. The top red line depicts the predicted suicide rates without fluoxetine and the bottom black line represents the actual rates with fluoxetine. (C) This figure demonstrates the linear relationship between suicide rates and fluoxetine prescription numbers.

**Figure 2:**
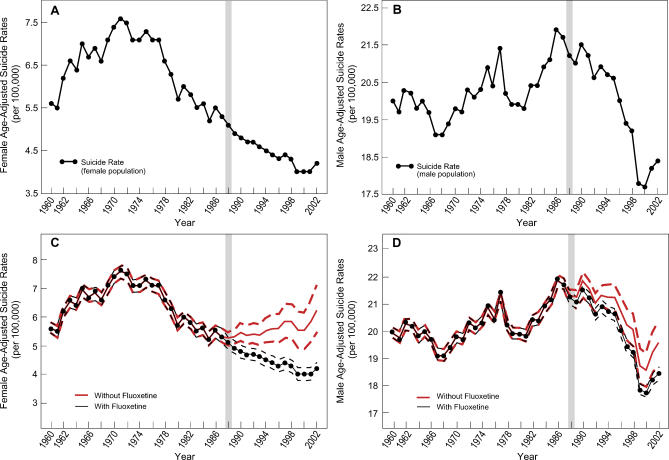
Age-Adjusted Suicide Rates by Sex (A and B) Suicide rates (per 100,000) for the total female (A) and male (B) populations from 1960–2002. (C and D) Age-adjusted suicide rate predictions for the female (C) and male (D) populations. Solid lines trace out the posterior median model predictions and the dashed lines depict the 95% Bayesian credible intervals. The top red line depicts the predicted suicide rates without fluoxetine and the bottom black line represents the current rates with fluoxetine.

Suicide rates had been fluctuating between 12.2% and 13.7% per 100,000 for the entire population from the early 1960s until the late 1980s, when they showed a gradual decline below 12%, with the lowest value of 10.4% in 2000 (Figure 1A and Table 2). For the male population, the rates of suicide had been higher, fluctuating between 21.9% and 19.1% in the pre-SSRI era, with a gradual decline since the late 1980s to their lowest value of 17.7% in 2000 (Figure 2B and Table 2). Likewise, suicide rates in the female population had fluctuated between 7.6% and 5.2% in the same period, showing a significant decline in the post-SSRI era at levels below the 5% mark in 1989, and they remained at 4% in the 1999–2001 period (Figure 2A and Table 2).

### Association between Suicide and Fluoxetine Dispensing

The steady decline in suicide rates for both men and women is associated with an increasing number of fluoxetine prescriptions from 2,469,000 in 1988 to 33,320,000 in 2002. A cross-correlation analysis of fluoxetine use and suicide rates in the period 1988–2002 shows a significant negative correlation: *r_s_* = −0.92, *p* < 0.001 (Figure 1C).

### Modeling Suicide Rates in 1988–2002 Based on Pre-1988 Trends

Under our time-series regression model, we estimate that the fluoxetine effect size β is −4.7 (posterior median; 95% Bayesian credible interval [BCI], −6.2 to −3.1) per 100 million prescriptions. Since the entire 95% BCI falls below zero, the data offer strong support for the hypothesis that fluoxetine decreases suicide rates. We also estimated that *ρ* = 1.0 (0.52 to 2.6), suggesting that nonstationary drift not caused by increasing fluoxetine usage remains in the underlying suicide rate trend, supporting the use of a nonstationary model.

In Figure 1B, the model estimates and predictions of age-adjusted suicide rates are plotted over time. Shown in black are posterior median and 95% BCI estimates incorporating the observed fluoxetine prescription levels for the years 1960 through 2002. In red, the plot predictions of suicide rates are superimposed on the number of fluoxetine prescriptions in fixed to 0 from 1988 onward. Beginning at year 1993, the BCIs with and without fluoxetine diverge, reinforcing the conclusion that fluoxetine is significantly decreasing suicide rates, even after controlling for potential drift in the underlying random walk. In terms of this drift, suicide rates between 1998 and 2000 decreased substantially more than would be predicted by increasing fluoxetine usage alone and are again seen to raise post 2000. It is unknown what is causing these additional dynamics.

Figure 2C and 2D plot the model estimates and predictions stratified by gender. Distances between observed suicide rates and predicted rates without fluoxetine are greater for females than for males. This observation is also reflected in our estimate of the differential fluoxetine effect size Δ. The posterior median estimate of ρ is −2.7 and its 95% BCI covers −6.4 to 0.92, with approximately 93% of its posterior mass lying less than zero. The zero point represents no differences between females and males. Several studies have shown that women are prescribed more antidepressants than men [37]; however, the data we utilized are not available grouped by gender throughout the study period. Therefore, our results suggesting that fluoxetine is more strongly associated with decreasing suicide rates in females than in males have to be interpreted with considerable caution.

**Figure 3:**
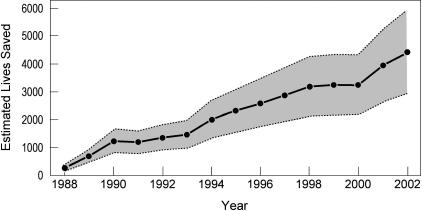
Estimated Number of Lives Saved Since 1988–2002 with the Advent of SSRIs Data are shown as a posterior median prediction with 95% Bayesian credible intervals.

Using the total number of observed suicide deaths (with fluoxetine) per year and our time-series model, we estimated the approximate number of additional deaths we might have expected if pre-1988 trends were maintained. If the hypothesis that fluoxetine decreases suicide rates were to be accepted, then taking the difference between the predicted and actual suicide deaths would estimate the number of suicides that were presumably prevented by SSRIs such as fluoxetine. Figure 3 shows these estimates for each year (posterior median prediction with 95% BCIs). Summing of these values in the period 1988–2002 would, on that basis, result in an estimation that SSRIs may have saved 33,600 (22,400–45,000) lives since their introduction.

## Discussion

A debate on the association of antidepressants and suicidality has intensified in recent months. Two possibilities exist: either antidepressants increase suicidality or by effectively treating depression, which is the principal cause of suicide, antidepressants reduce suicidality. It is important to note that there is a substantial difference between suicidality, which is defined by feelings, thoughts, and behaviors related to suicide, and actual deaths caused by suicide. While suicidality is alarming, we believe that the actual impact of antidepressants could best be assessed at the public health level by a study of actual numbers of completed suicides.

Grunebaum et al. [38] previously reported a temporal association between increased use of SSRIs with decreased rates of suicide in the US in 1985–1999. In concordance with the findings of that study, we found here a highly significant negative correlation between fluoxetine dispensing, which is predominantly used to treat major depression, and US suicide rates in the period 1988–2002. We also modeled suicide rates in the period post-1988, using historical data to better represent the underlying random walk that rates take over time. While overall mortality rates have not decreased since 1988, suicide mortality has decreased steadily since 1988. Our modeling analysis shows that if pre-1988 trends (pre-SSRI era) were extrapolated through to 2002, mortality numbers by suicide would have been higher by approximately 33,600 cases. This could lead one to hypothesize that SSRIs have saved approximately 33,600 lives in the United States alone during this period.

Our modeling results also suggest that fluoxetine has been associated with a greater decrease in suicide rates in females than in males. This finding is based on a model that assumes that affected females and males are equally likely to be prescribed and take fluoxetine. However, further studies are warranted to examine and further define this modeling assumption and to further investigate the prescription rates for males and females, including compliance and follow-up issues between the two sexes. If females were more likely to seek treatment for depression and follow through with the drug regimens and therapy course than males, they would benefit more from SSRI use. This might explain the greater demonstrated drop in rates of suicide for the female population. Unfortunately it is not possible to look at fluoxetine dispensing by sex, as such data are not available from IMS or from other sources. An alternative and not mutually exclusive explanation could be based on the fact that antidepressants are frequently used in overdose as method for suicide attempts. A shift away from older, far more lethal in overdose, tricyclic antidepressants to SSRIs could therefore have some impact on the reduction of suicide rates. This impact could be greater in females, in whom intoxication is by far the most often chosen method for suicide attempts.

Similar to what has happened in the US, in many other developed countries the numbers of prescriptions for SSRIs such as fluoxetine have increased steeply since their advent in the late 1980s and early 1990s. A study looking at changes in suicide rates for the Australian population and exposure to SSRIs for the 1991–2000 period also found a significant association between the two. They found that the higher the population's exposure to antidepressants, the larger was the decline in rate of suicide. Most significantly, this was demonstrated in the older age groups, in which rates of suicide decreased substantially in association with exposure to SSRIs [39]. Similarly, in Sweden, which has a higher total population suicide rate, prescription rates for SSRIs increased dramatically in the early 1990s, and the rates of suicide showed an inverse relationship to the rates of antidepressant prescriptions in most age and sex groups [40]. In another study in Sweden, which examined official mortality statistic data for 1977–1997 and data on antidepressant use from surveys of sales to pharmacies, they found that suicide rates in general declined over the whole study period, but the rate of decline accelerated after the introduction of SSRIs in the early1990s [41]. In yet another European study in Hungary for the period of 1984–1998, antidepressant prescription rates rose steeply after the introduction of SSRIs in the early 1990s. With this, the rates of suicide proportionally declined, despite steep increases in unemployment and per capita alcohol consumption [42].

Recent studies on antidepressant-related suicidality [43–45] did not address the effects of antidepressants in the overall population over the long term and examined only the acute outcome measured for the treating drugs in question. In contrast, we looked at the possible impact of SSRIs over a 14-y period and the resulting cumulative overall effect on suicide rates over that period. In the present study we tried to address the issue of whether acute and transitory thoughts and feelings of suicide related to antidepressant treatment were in the long term associated with increased suicide rates, or if the opposite might be occurring: by effectively treating depression, antidepressants could be associated in a reduction of actual suicide rates.

Our results indicate a temporal association between the introduction of fluoxetine and increased dispensing patterns and decreased rates of suicide, but they do not establish a mechanistic cause. When the competing hypotheses (i) that antidepressants can trigger suicide, or (ii) that by treating depression antidepressants reduce suicide rates, are examined in light of our results, it is logical to refute the concept that on a population level fluoxetine dispensing is associated with increased suicide, because not only did suicide rates start to decrease after fluoxetine came to market, but we also show here a highly significant negative correlation between fluoxetine dispensing and suicide rates. If the opposing hypothesis (i) were true, then we would have expected to find an overall rise in the rates of suicide after the introduction of SSRIs or at least not a drop in the rates. Moreover, according to our modeling, if pre-1988 (and hence pre-fluoxetine) suicide trends had persisted to the end of the study period, the number of suicides would have been in the range of 33,600 higher in cumulative terms.

A limitation of this work is that the analysis was across combined age groups. This could not be helped for the prescription data, which is not categorized by age, but the suicide data are available by age (and by sex). Gunnell and Ashby [46] found that there were very different trends in suicide across different age groups, which could complicate the interpretation of our analyses. In the absence of prescription data by gender and age across the entire study period, we felt it would less problematic to simply analyze the data by sex of suicide victims than to also introduce another variable that would lead to complex stratifications by various age cohorts in both sexes in the absence of actual prescription data by age group.

In this study we used fluoxetine as an index of SSRI use. It was the first and most widely used of this class of drugs. Would our results have been different if we had included data on all SSRI prescriptions in the United States since 1988? If we assume that all SSRIs are equivalently effective and group them together, then the structure of our Bayesian model does not change. What does change is that the number of prescriptions, particularly in recent years, would be higher than reported in this analysis. Furthermore, given our hypothesis, we would expect that this correlation would only increase with a larger number of prescribed SSRIs other than fluoxetine. In Figure 1C, this modification would stretch the far right values further to the right, suggesting a stronger correlation between antidepressant use and decreased suicide numbers. Under the equivalency assumption and by restricting our analysis to fluoxetine, we present here a conservative estimate of the strength of the negative correlation between antidepressant use and completed suicides. Natural extensions to the linear regression portion of our Bayesian model exist to test for differences between the effectiveness of various SSRIs at the population level.

As elegantly stated by Gibbons and colleagues [47], it should be noted that causal associations cannot be established with this type of observational data. Moreover, we would like to caution that this type of data and study is limited by its large scale, and does not examine specific effects on smaller parts of this population. Therefore, although SSRIs would seem likely to have significant public health benefits in decreasing suicide rates, with this type of data we cannot confidently rule out the possibility that fluoxetine treatment is associated with an increased risk of suicide in a smaller group of individuals. However, we can conclude that, for the entire US population, a direct, inverse correlation exists between suicide rates and fluoxetine, and that treatment with fluoxetine (or possibly all SSRIs) for depression and other mood disorders may have contributed to the prevention of as many as 33,600 suicides since the drug was introduced.

Similar to our results in adults, a recent study by Weller et al. [48] in the US, showed that the past decade has seen a significant drop in the rates of adolescent suicide, which coincided with the onset of the use of these SSRIs. Those authors cautioned that a reduction in the use of SSRIs in children and adolescents should therefore be considered very carefully.

Our results support the hypothesis that at a population level, SSRI treatment is temporally associated with decreased suicide rates, presumably due to effective treatment of major depression, which when untreated is the principal cause of suicide. If this conclusion is correct, decreased rates of antidepressant dispensing could have the opposite effect of leading to increased suicide rates. Current trends of decreased antidepressant sales are therefore worrisome. We suggest here that public health efforts to address suicidality caused by acute antidepressant treatment should be tempered by an awareness of our data showing that at the population level antidepressant use is highly negatively associated with actual deaths by suicide in the long term. Consequently, limiting the use of antidepressants may eventually result in increased deaths by suicide. While our study was based on the prototypical SSRI fluoxetine and this type of study is limited, future studies could further expand on our results to examine other SSRIs and data from other countries, both developed and developing. Finally, although the current issue concerning antidepressants and suicidality requires further examination, we believe that many more lives have been saved than lost since the advent of these drugs.

## Abbreviations

AR: autoregressive
BCI: Bayesian credible interval
CDC: Centers for Disease Control and Prevention
SSRI: selective serotonin reuptake inhibitor
